# Sustainable Resumption of Cardiac Catheterization Laboratory Procedures, and the Importance of Testing, During Endemic COVID-19

**DOI:** 10.1007/s11936-021-00901-w

**Published:** 2021-02-22

**Authors:** Mahvash Zaman, Denise Tiong, Jacqueline Saw, Sarah Zaman, Matthew J. Daniels

**Affiliations:** 1 Manchester Heart Centre, Manchester Royal Infirmary, Manchester University NHS Foundation Trust, Manchester, UK; 2 Division of Cardiology, Vancouver General Hospital, University of British Columbia, Vancouver, BC Canada; 3 Westmead Applied Research Centre, University of Sydney, Sydney, Australia; 4 Department of Cardiology, Westmead Hospital, Sydney, Australia; 5 Division of Cardiovascular Sciences, Manchester Academic Health Sciences Centre, University of Manchester, Manchester, UK; 6 Division of Cell Matrix Biology and Regenerative Medicine, University of Manchester, Manchester, UK

**Keywords:** COVID-19, Cardiac catheterization laboratory, COVID testing, Vaccine, Sustainability

## Abstract

**Purpose:**

As second and third waves of the COVID-19 pandemic challenge healthcare in North America and Europe once again, we analyze the impact of the first wave on routine elective cardiovascular care, and the differential COVID risk emerging within our patient groups and staff.

**Perspective:**

We describe the need to sustainably resume, and temporarily expand, routine elective cardiac services in the face of resurgent COVID-19. Some, but not all, cardiac patient groups are particularly vulnerable to adverse outcomes following COVID-19 infection. We explore mitigation measures at the institutional level to increase resilience within cardiac services to enable them to operate deep into subsequent waves of COVID infection which place unprecedented demands on intensive care infrastructure. As measures to eradicate the virus appear to have failed in many countries, and vaccine roll-out will take many months we take the view that the threat imposed by endemic COVID-19 alters the way elective procedural care should be offered to cardiovascular patients.

**Conclusion:**

Our patients are at definite risk from their cardiovascular disease, and a return to suspension of proven prognostic interventional treatments on an elective basis – the default for the first wave – must be avoided at all costs.

## Introduction

The emergence of SARS-CoV-2 triggered a global pandemic which challenged society in general and healthcare in particular. Limited testing capacity, absence of proven treatment and exponential community transmission led to an initial tsunami of COVID-19 patients with resultant public healthcare restrictions and healthcare measures. COVID-19 is now endemic in many countries, with ongoing potential for repeat waves of infection. This article explores the operational aspects of delivering routine elective institutional cardiac care in the face of the persisting threat of COVID-19.

### COVID-19 impact on volume—we now need to do more, but can only offer less

Cardiac catheterization laboratory (CCL)-based procedures were routinely offered pre-COVID-19 for investigation or treatment. Most therapies have prognostic or symptomatic outcomes that impact on the duration or quality of life for patients. Many cardiac procedures are elective in nature but offered in a timely fashion. A proportion is time-critical, such as PCI for myocardial infarction, and life-saving. With uncertainty at the pandemic onset, and the saturation of hospitals in Milan (Italy) and New York (USA), many healthcare services suspended elective CCL procedures. Only those with a clear time-critical component remained. Patients experienced unprecedented delays in routine cardiac care. However, the unintended consequences of such stark disruption were significant declines in time-critical conditions themselves. ST elevation myocardial infarction (STEMI) fell ~ 24–40% across the USA, Europe, and China [1–5], while emergency pacemaker implantations reduced by 73% [6]. Naturally, at an institutional level, declines have many causes. Patient factors, such as fear of COVID-19 exposure [7], combine with institutional factors following internal restructuring of acute services, redeployment of diagnostic capabilities, and staff sickness due to COVID-19 itself. Inevitably, an avoidable loss of life accounts for a proportion of the excess deaths of the first wave of the COVID-19 pandemic. New York, for example, experienced a large increase in non-respiratory deaths, the majority attributable to heart disease [8•].

One clear challenge in resuming routine CCL activity is handling the displaced volume of routine work that accumulated, while elective services were curtailed. This catch-up needs to occur in conjunction with limiting person-person transmission of COVID-19 in the healthcare setting. These measures directly reduce CCL efficiency. Additionally, we face the challenge of late-presenting pathologies which follow interruption of diagnostic, outpatient, and primary care services. By April 2020, 1 month into the UK lockdown phase, more than 195,000 people were waiting for cardiac investigations or procedures. During the pandemic phase, NHS England figures report a 67% reduction in echocardiography, with primary care referrals to cardiology a quarter of pre-pandemic levels [9]. Cumulative delays in diagnosis and treatment may produce long-term cardiovascular complications and mortalities potentially preventable by earlier treatment, providing an impetus to resume, and sustain, cardiac services even in the face of resurgence of COVID-19.

In summary, the acute effect of COVID-19 on the CCL was a sharp reduction in elective and emergency procedural activity. The extent to which patients identified for CCL based procedures have been deferred may vary between institutions, but as the current situation in Southern California [10, 11] reveals, the opportunity to treat them between waves of COVID infection may be fleeting. Patients will not present, even with life-threatening conditions, if they perceive their risks are higher within a hospital than at home. Advanced pathologies not seen for decades may return to clinical practice due to cumulative delays upstream of the CCL. Cath lab volumes need to temporarily increase to catch-up, but operational policies to minimize infection dictate how this can be achieved—e.g., 7-day working, extended hours—as the routine model of 9 am–5 pm care pre-COVID-19 can only return when COVID infection rates are low.

### Does the shadow of COVID-19 fall equally on resuming all procedures in all patients?

The acute impact of COVID fell unevenly in the CCL. Emergency procedures that acutely save life (e.g., pacemaker for complete heart block) were prioritized over elective procedures that save life in the longer term (e.g., primary prevention ICD). Attempts to sustain activity in elective structural heart disease were rationalized in the context of patient symptoms, anatomy, and prognosis [12]. However concomitant COVID infection often dominated the clinical outcome [13] with mortalities an order of magnitude higher than pre-COVID trial and registry data predict. Anticipated operational challenges to emergency care [14] were also apparent; for example, emergency STEMI care saw an ~ 11% increase in symptom to hospital time, 20% increase in door to balloon times and 20% adoption of thrombolysis [15•, 16, 17]. As we restore elective activity, we must learn from the first wave and identify which procedures, or patients, might have more, or less, risk from nosocomial COVID-19 infection during hospitalization. By extension, this reveals which services should remain in the event of subsequent waves of infection. The first challenge is how to get going again.

Poulin and Pinto [18] outlined strategies to facilitate CCL resumption. They suggest a phase-in based on categorizing elective patients according to symptoms, procedure type, and institutional factors. A consensus North American cardiovascular societies guidance document suggests reintroduction of all invasive and diagnostic cardiovascular procedures according to their risks with sustainability altered in response to the severity of the pandemic [19]. With resumption underway, the pressing challenge of the moment is how to adjust, and sustain, recovery in the face of resurgent infection. Prioritization driven by patient symptoms alone may be misguided as it denies prognostic interventions, like primary prevention ICD, to young asymptomatic patients.

Age, followed by male sex, is clearly the dominant risk factor for adverse outcomes (hospitalization, organ support, and death) following COVID-19 infection [20]. Yet CCL procedures are offered across a broad age range, from the pre-term neonate to the centenarian. In the UK, for example, the average age of patients treated with trans-catheter aortic valve replacement (TAVR), percutaneous coronary intervention (PCI), or percutaneous closure of the foramen ovale (PFOc) is 83, 65, and 45 years respectively, spanning the age spectrum of COVID-19 risk. Furthermore, hypertension, diabetes, coronary artery disease (CAD), and obesity are the most common comorbidities in inpatients with COVID-19, with obesity, diabetes, and CAD over-represented in fatalities [21••].

Analysis of PCI for stable angina outcomes during the first wave of the pandemic revealed non-cardiac causes of death dominate 30-day mortality, a large component of which is attributable to COVID-19 infection [22]. Whether these patients acquired COVID during hospitalization or in the community is unknown. The association between severe COVID illness resulting in death and CAD, or conditions predisposing to CAD, highlights the need to consider this dimension as an aspect of these patients’ optimal care. Adjustments may include pre- and post-procedure isolation, and perhaps procedure deferral, particularly where prognostic indications are known to be lacking (e.g., elective PCI for stable angina, or elective catheter ablation of arrhythmia), in patients who are particularly vulnerable (e.g., older, male, comorbid patients), particularly when community COVID rates are rising and nosocomial COVID transmissions occur.

Finally, we should consider the procedure itself and recognize the competing needs at an institutional level. In private healthcare systems, revenue streams need to recover, but different procedures have different tariffs. In teaching hospitals, training has been disrupted, but curricula need to be completed. In all settings, waiting lists have grown and need to be reduced, but different procedures require different amounts of CCL time. If CCL time is limited, should institutions defer time-intensive procedures (e.g., chronic total occlusion PCI), and instead have initial preference for shorter duration procedures? Procedures place variable demands on the hospital estate. The adoption of minimalist practice and same-day discharges, where possible, may limit potential for nosocomial transmission, and preserve a service when competing needs driven by a second wave occur. The spring peak of the pandemic consumed ICU capacity, with 10% of COVID-19 patients requiring intensive care [23]. Ideally restructuring cardiac services between waves may generate resilience at the institutional level to accommodate fluctuating COVID-19 attributable demand—which previously curtailed all non-urgent cardiovascular diagnostics and interventions [24, 25]—without restricting hospitals to a “COVID-only” service.

**Fig. 1:**
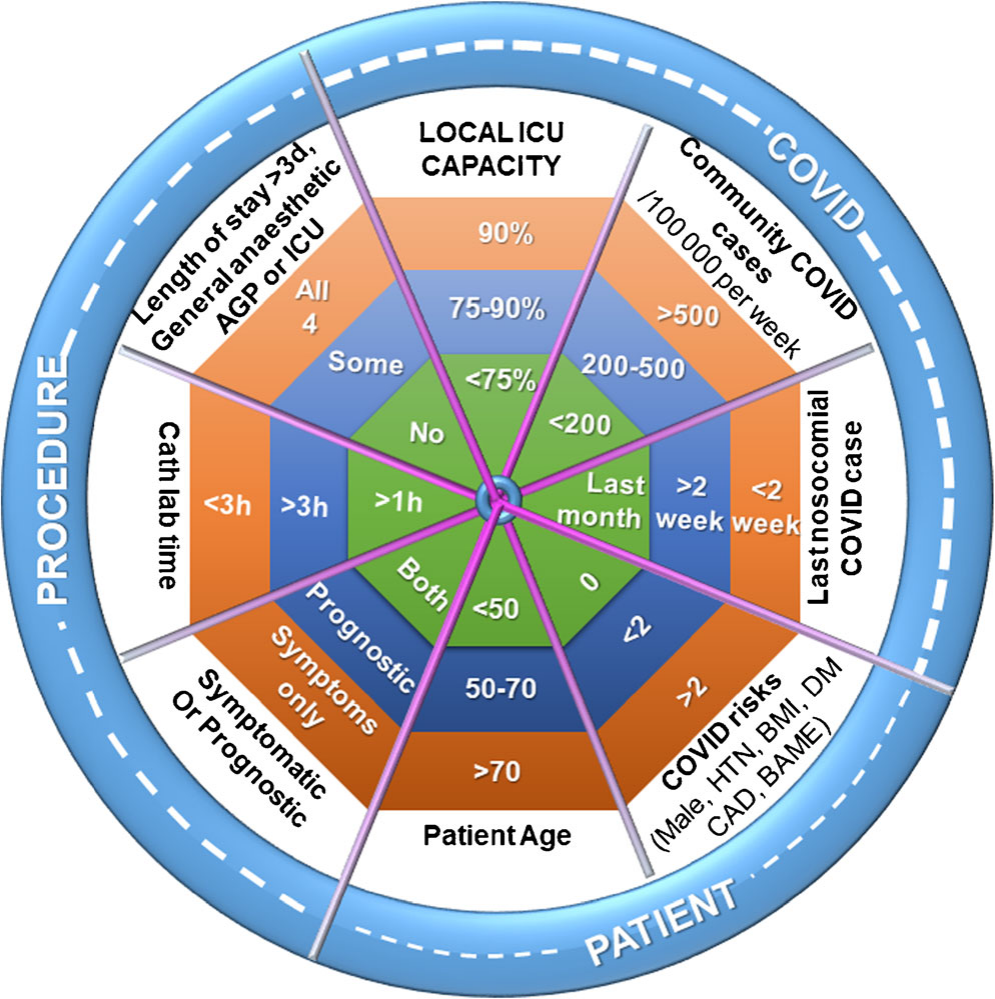
Resuming and sustaining elective catheter lab activity during endemic COVID-19 depend on competing demands of the procedure, patient vulnerability, and COVID-19 transmission rates. Safer profiles are in the green zone; adverse profiles are in the orange zone. Five hundred COVID cases typically generate ~ 50 hospital admissions, of which ~ 10 will need ICU admission for ~ 10 days. Public health measures to control the virus take several weeks to have an effect because of the long pre-symptomatic phase. To balance the competing interests of public health, with institutional and operator demands, we suggest that that green tiles should always outnumber orange tiles when determining elective case scheduling. Institutions may wish to set their own thresholds based on local population demographics, which will impact COVID-19 severity, and ICU surge provision. HTN = hypertension, BMI = body mass index, DM = diabetes mellitus, CAD = coronary artery disease, BAME = Black, Asian, minority ethnic, AGP = aerosol-generating procedure, ICU = Intensive Care Unit.

In summary, the CCL exists in a complex landscape. We need to reconsider priorities continuously as the tide of COVID-19 rises and falls [Fig. 1]. The landscape has multiple aspects derived from the patient, the procedure, and the prevailing COVID-19 situation. Rapid, day-case procedures for young patients which are supported by prognostic data should continue well into a COVID wave because the COVID-associated risks are small, and the procedure-associated benefits are large. Resource intensive procedures for older, co-morbid patients offered for symptoms rather than prognosis are perhaps better deferred, because the risks of COVID-19 massively outweigh the potential procedure benefit. Finally, in order to build resilience into the hospital system, the CCL faces a downward pressure to maximize patient treatment while minimizing demand on the hospital estate. Many CCL procedures have demonstrated equivalent clinical outcomes, while offering reduced length of stay, compared to traditional open-heart surgical alternatives. Redistributing activity from cardiac theaters to the CCL will necessarily displace other activities. Diagnostic services with non-invasive alternatives appear to be most vulnerable. Reducing multiple attendances by adopting a diagnose ± treat approach, may be applicable beyond PCI but require different standards in clinical governance. Intra-cardiac echocardiography ± PFOc, to detect and close a PFO, could substitute for trans-esophageal echocardiography (TEE) to diagnose and then subsequently guide PFO closure either side of a multi-disciplinary team discussion. This condenses two aerosol-generating procedures (AGPs), into a single non-AGP hospital visit.

### Resuming elective services in a COVID endemic era: pillar 1—testing

Sustainable resumption of elective catheter procedures may be supported by four pillars at the institutional level. Having successfully conveyed the public health message of the risks of COVID-19, there is now the challenge to restore non-COVID health services for patients who declared a reluctance to attend hospital, even with life-threatening emergencies.

As symptoms alone appear to be insufficient for case identification [26], COVID testing is central to the sustained return of elective procedures. Testing patients and staff limits the potential for asymptomatic staff to infect patients, inadvertent patient-to-patient transmission, and elective procedures in pre-symptomatic COVID patients who may have a COVID dominated post-procedure outcome. Maintaining COVID-free environments within the hospital propagates the message that elective treatment is safe and is vital to prevent excess mortalities from COVID-19 infection in the typical cardiology patient cohort. Testing allows ward-based zoning of patients according to COVID status, reducing the possibility that patients screened negative and isolated ahead of elective procedures will be inadvertently exposed to COVID-19 by emergency admissions who do not have the luxury of enhanced quarantine [27]. The transmission vector between patient groups is potentially the staff who works across the hospital site. Some institutions have initiated regular staff testing to limit this route of nosocomial spread [28]; the optimal frequency and utility of this approach are yet to be demonstrated.

Current COVID-19 testing uses reverse transcription-polymerase chain reaction (RT-PCR) of the viral genome and takes 4–6 h to run, with logistics extending the turnaround time. This makes it unsuited to time-critical patient screening but potentially suitable for elective screening when conducted close to the procedure. A variety of RT-PCR-based screening platforms, differentiated by trade-offs between scale, speed, and accuracy, are available. These include the Roche cobas SARS-CoV-2 assay, Cepheid Xpert Xpress SARS-CoV-2, and more recently, the CovidNudge which, respectively, take 3.5 h, 45 min and 90 min to run [29, 30]. To facilitate quicker diagnostic testing, applicable to time-critical patients, the Abbott ID Now SARS-CoV-2 assay was developed, which returns a result in 5 min. A rapid result is not always an accurate one, and in head-to-head comparison, this platform had a lower sensitivity than the conventional assay, mainly driven by failure to detect at low viral load [29]. Regardless of the assay platform used, the need to attend hospital for testing seems counterproductive. Hospitals will attract COVID-19 cases from the community. Patients may avoid hospitals if community rates are much lower than healthcare environments. Hospitals should do everything possible to be COVID-free and establish decentralized testing in order to sustain the range of elective treatments or investigations they offer.

RT-PCR testing is not perfect. When infection rates are high, a major limitation is the rate of false-negative tests. Approximately 2–33% of patients with COVID-19 have a false-negative result [31]; hence, isolated testing in the absence of strict quarantine for elective procedures may have limited impact. Equally, when infection rates are low, a significant proportion of positive results will be false positives [32]. Even in symptomatic patients, typical testing programs identify < 10 infections per hundred screened. Infection rates at a population level are much lower, often quoted per 100,000 population. With infections at low levels, the costs of testing (> $100/test) are not insignificant. Accepting that population level testing and tracing may be the only route to reopen society until a vaccine has been widely deployed, and acknowledging that it may also be part of the argument to convince patients that hospitals are safe places for treatment, it appears that many of the criteria established for a screening test [33] are not met. Cost effectiveness reporting for COVID screening protocols has yet to be determined. Hospital administrators, staff, and patients will naturally question the utility of a test that changes the outcome for a small number of positive results, which complicates the pre-admission process for all. Screened negative patients, and their families, may be less sympathetic in the event of nosocomial COVID infection if they do not understand that these measures reduce, but do not eliminate, the possibility of transmission of an endemic infection.

As the northern hemisphere reaches winter, we must recognize that symptoms cannot distinguish seasonal viral infections (with low case fatality rates), from COVID-19, with high case fatality rates. This increases the need for accurate and timely testing. The ideal test would resemble the common pregnancy test which can be performed outside a healthcare setting, at lower cost, and complexity. Such tests are generally based on the presence of protein antigens. Abbott’s BinaxNOW™ COVID-19 Ag Card obtained FDA Emergency Use Authorisation in August 2020 [34] and appears to have this potential, although the clinical impact is uncertain at the time of writing. Sensitivity and specificity of the test appear good enough to be useful (positive agreement with RT-PCR 97.1% (95% CI: 85.1–99.9%); negative agreement: 98.5% (95% CI: 92.0–100%), although the limitation posed by low viral load on the swab remains. The low cost and low complexity contrast sharply with the infrastructure demands of RT-PCR, but the corollary to decentralizing testing away from dedicated laboratories is the lack of infrastructure to record and report the test result—either within the hospital system or in the wider public health context.

In summary, testing of staff and patients appears to be the most robust pillar to sustain procedural activity. It is highly important because COVID-19-infected individuals are infectious before they become symptomatic. Centralized testing capabilities are the most accurate but are also costly and slow. Antigen-based point of care testing has recently been released but not yet become embedded in the wider matrix of reporting, and the extent to which it delivers decentralization (e.g., testing in pharmacies, primary care, or nursing homes) remains to be proven. The hallmark of a successful testing program paradoxically is the number of negative results. Health economic analyses of COVID testing in many domains are needed.

### Resuming elective services in a COVID endemic era: pillar 2—immunity and vaccination

The role of antibody testing to identify those previously infected remains unclear. There is no strong evidence to demonstrate that those with a prior infection develop long-lasting immunity. Antibody levels rise following infection; IgM antibodies are detectable 5–10 days from symptom onset, followed by increasing IgG antibody concentrations. Seroconversion takes approximately 40 days to complete [35, 36]. Serology testing is therefore not a useful marker for early infection. Although immunity passports [37] have been proposed, whether the presence of antibodies confer protection remains unclear. Some studies show raised IgM antibody concentrations correlate with poorer outcomes, hence suggesting that having antibodies is not necessarily a marker of protection [36]. Indeed, reinfection has been documented several times, including a case where the second infection was clinically more severe than the first [38]. If this is generalizable, the impact of prior infection and vaccination efforts may be difficult to predict.

Population immunity can be achieved via natural infection or through vaccination. Achieving immunity through natural infection alone is predicted to be accompanied by an unacceptable death toll predicted to exceed 30 million people globally [39]. The World Health Organization (WHO) reported 42 candidate vaccines in clinical evaluation, with ten in late-stage trials as of the 2 of October, 2020 [40]. The first vaccine approved for routine clinical use was Sputnik V, after the Ministry of Health in Russia approved it in the absence of phase 3 trials raising concerns of safety and efficacy [41]. Subsequently trial data and regulatory approval for the synthetic RNA Pfizer-BioNTech BNT162b2 vaccine encoding the SARS-CoV-2 full-length spike protein in the pre-fusion conformation [42] have been widely granted and rapidly brought to clinical practice with over 2 million patients having received the first dose of the vaccine to date. Regulatory approvals are anticipated for other vaccine strategies with positive phase III outcomes. The exceptional efficacy (> 90%) demonstrated by these agents is potentially transformative for healthcare. As a minimum, it should protect the workforce as severe infection is reduced. It should also enable “high-risk” personnel to return to the front line.

There is present uncertainty about whether the vaccine prevents asymptomatic carriage and transmission. If this does happen, nosocomial transmission by staff to patients may be possible to eliminate. Zoning of patient groups would become much more robust, benefiting the CCL tremendously as patients move in and out of this quantal facility from all areas of the hospital.

Inevitably uncertainties exist with key questions related to efficacy of combination vaccine regimes, durability of antibody response, the unknown potential for future side effects, and vaccine safety in groups excluded from vaccine trial populations. Particularly challenging will be the messages to overcome anti-vaccination rhetoric which may hold greater sway in some of the population, who may decline vaccination and in doing so prevent the rapid acquisition of herd immunity that can lead to virus eradication. As has been the case for other viruses, SARS-COV2 will naturally evolve over time [43], and over decades, SARS-COV2 may attain the same impact on society as the other corona virus family members that cause the common cold. However, in the short term, it is possible that with millions currently infected worldwide, enough variation has been established in the existing viral genome pool over the past 12 months to allow one (or more) strains to spread in spite of the current vaccine regimes.

If we consider the response to the pandemic in terms of technologies developed, a familiar pattern for any infectious disease is apparent. The first challenge in the COVID-19 response was to establish diagnostics to identify those infected. In the CCL, this was largely adopted as a test to identify those not infected ahead of elective treatment. Yet proving a negative is difficult—particularly when it is impossible to see the virus in order to collect it with a swab. The second milestone has been to develop, and deploy, effective vaccines to the population at large. The third, and currently largely unmet need, is to be able to measure neutralizing antibodies to COVID-19 [44]. In the CCL, this would allow us to stop the error prone screening strategy for the absence of infection, but rather test for the presence of something that makes infection impossible.

### Pillar 3: back to basics and back to the future—barriers, distance, risk, and telemedicine

Social distancing, hand hygiene, mask wearing, and meticulous cleanliness within the built environment reinforce the public health measures that our patients and their families expect to see. They help employers ensure the safety of their staff. It is notable that in the early analysis of UK healthcare worker deaths from COVID-19 [45], no intensivists or anesthetists died, even though they were in an AGP environment with many of the sickest COVID patients. This suggests that the combination of enhanced PPE, staff/patient ratios, and built environment offered in the ICU might be the optimal strategy for protecting the workforce more generally. As we anticipate further waves of COVID-19 until population level vaccination is well underway, hospitals need to ensure supply chains of PPE, and consider extending an ICU-like model of care within the hospital with reduced patient density, particularly for cardiac services that sustain the activities in the CCL which must keep running to prevent the avoidable loss of life that follow suspension of elective prognostic procedures seen in the first wave.

Equally important as the recognition that the risks of COVID-19 bear down differently in certain patient groups is the realization that COVID-19 risks are asymmetric within the workforce. NHS employers and the British Medical Association have published guidance and risk assessment tools to help organizations identify vulnerable healthcare workers [46]. A disproportionate link between Black Asian and Minority Ethnic (BAME) is well established. UK national audit data shows that ~one-third of COVID-19 patients who needed critical care admission were from BAME backgrounds [47]. Analysis of COVID-19 mortality data among NHS staff demonstrated that those of BAME origin formed the majority of deaths (64%) [45]. The Office of National Statistics (ONS) found Black ethnicity patients had ~ 3.5× the risk of dying from COVID-19 compared to white counterparts. The Association of Local Authority Medical Advisors (ALAMA) has devised the COVID-age tool to assess an individual’s risk and to help manage staff return to work [48]. Targeting vaccination to at-risk groups should be considered.

Pregnant CCL staff may also represent an at-risk population with considerations for both mother and fetus. Pregnant women do not appear more susceptible to SARS-CoV-2 infection; however, more severe COVID-19 illness has been described, particularly toward the end of pregnancy [49, 50]. In regard to fetal risk, there has been no data linking COVID-19 to early pregnancy loss (miscarriage) or birth defects. However, concerns have been raised for the third trimester (> 28 weeks) due to higher rates of premature delivery and risk for maternal-infant transmission [51]. Another issue pertinent to healthcare workers is the use of N95 masks; pre-COVID-19, these were shown to compromise maternal cardiorespiratory function and fetal oxygenation during the later pregnancy stages [52, 53]. While more data is needed, during the pandemic phase of COVID-19, pregnant healthcare workers > 28 weeks have been advised to avoid direct patient contact [54]. Pregnancy was an exclusion criteria for the vaccine trials.

Extending social distancing principles within the built hospital environment exploits the limited ability of the virus to travel between patients. However, the ultimate distancing solution does not bring patients into hospital at all. Telemedicine enables ongoing patient care while reducing the risk of exposure for both health workers and patients. Furthermore, it saves patients time and money [55]. Telemedicine can be applied to routine follow-up or pre-operative assessments, obtaining consent and triaging patients who need to be assessed in person. It has also enabled staff who have to isolate to remain part of the care team, consequently freeing up colleagues from these duties. Telemedicine may be better than the traditional model; cardiac rehabilitation delivered in this way was associated with reduced hospitalizations and cardiac events compared to usual care [56]. However, the absence of a face to face consultation is not suitable for all cardiology patients, for example, those not familiar with electronic devices, or limited by presbycusis or cognitive impairment. Telemedicine does not allow physical examination, or routine outpatient diagnostic tests like ECG and echocardiography which often play central roles in the longitudinal follow-up of patients. Aside from identifying, and supporting patients pre- and post-CCL procedure, the role of telemedicine may extend into the CCL directly in the form of tele-proctoring [57]. Research may also benefit from the transition to remote telemedicine-based follow-up of study participants tracked via digital platforms measuring drug compliance [58] or physical activity.

### Resuming elective services in a COVID endemic era: pillar 4—human resources

Historical workforce planning assumptions for cardiology were made without the COVID pandemic in mind [59]. With no mandated age of retirement in the USA, nearly a quarter of physicians are over 65 years old [60]. The short-term measures to redeploy younger cardiologists that could face COVID patients, and shield those older, or medically at risk doctors that could not, raise many workforce dilemmas as many of us now face endemic infection without herd immunity or preventative therapy. How do we reintegrate those who were isolated back into the workforce? Even more provocatively, should we aim for full reintegration into the workforce if repeated waves of infection will occur every few months, and take many months to control? At what point will the “lean-in” efforts to support a reduced physician base accelerate burnout and lead to service failure among those who do not have to isolate? How do you escape the subconscious bias of age, gender, and race in recruitment which will inevitably have a COVID-19 angle to it, especially if recruitment is triggered by a COVID related pressure? To what extent do institutional “COVID-preparedness” and “COVID-resilience” strategies need to be a part of contractual terms of employment and recruiting strategy?

How many doctors do we need to provide a service? Historical staffing levels are predicated on the volume of activity related to a particular disease, or procedure, and projected models of utilization. This is often considered in the context of competing local providers and an institutional desire to invest or accelerate particular programs. Contemporary cardiology practice trends see fewer investigations, and interventions per physician encounter somewhat offset by the increase in the > 65 “baby boomer” generation, and rising obesity rates [61] with typical predictions for physician shortages on a national level. However, these assumptions are now challenged by COVID. As a profession, how do we fare if the volumes do not come back?

**Fig. 2:**
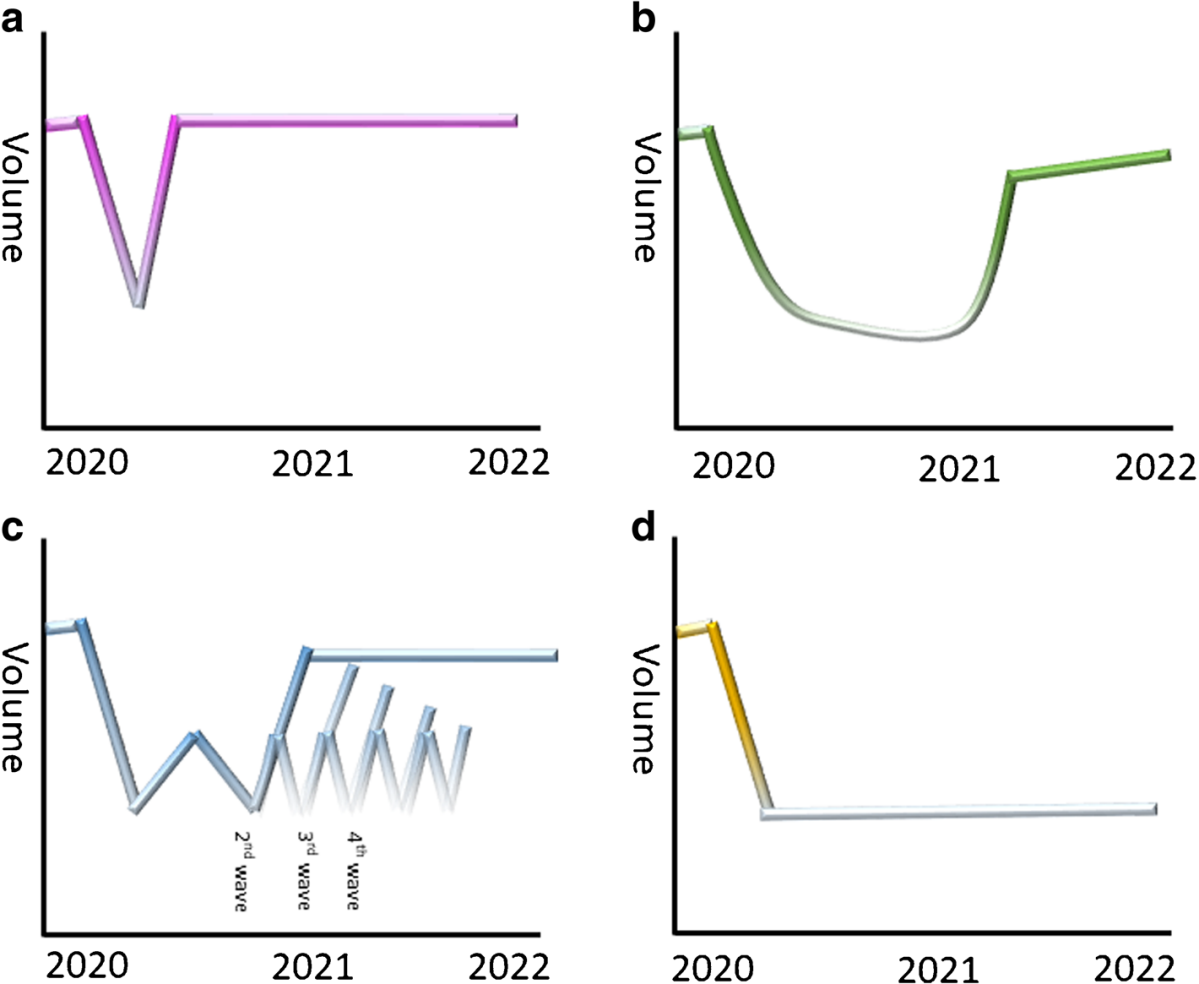
Elective activity may return differently within cath lab services. **a** “V recovery” following the initial lockdown is a rapid return to normal activity; this paradigm will apply to prognostic services offered to younger patients, e.g., congenital heart disease. **b** “U recovery”, this will apply where there is a prolonged inability to provide, or work-up, a particular patient group, or institutional preference for alternatives—e.g., non-invasive angiography. **c** “W recovery”, recurrent waves of infection suspend elective procedures. **d** “L recovery” the enduring absence of a particular procedure eg following service reconfiguration. The assumption that activity will return to pre-pandemic levels may not hold out, especially for procedures offered to patient groups with high COVID-19 mortality as fewer patients survive after each wave of infection (**c**).

The recovery scenarios for the economy at large, commonly summarized as “V, W, U and L”, may also play out variably in the CCL [Fig. 2]. With an average 9 years’ life lost per COVID infection [62], and over 360,000 deaths in the USA alone, it is possible that widespread COVID infection will significantly change the societal demographics used to estimate procedure volumes. This would be further compromised if the acute reductions in referrals seen early in the pandemic were to persist into the recovery phase as patients present late for investigation or management, or even succumb to infection. A more destabilizing model arises if telemedicine facilitates assessment of patients outside their typical referral networks. This could be driven by adverse publicity related to nosocomial COVID transmission in an institution, pandemic-related delays in treatment or local COVID infection rates which consume ICU capacity and halt elective programs. If patients begin to explore their treatment options over larger geographical ranges, the physician workforce may have to follow.

Another aspect of workforce planning is job satisfaction. Interventional cardiologists typically derive personal reward from the procedural aspects of the job, which is often indexed to reimbursement. In centralized healthcare systems, the institutional desire to have an agile physician workforce that can be redeployed to overwhelming COVID demand will be at odds with the specialist aspirations, which drive physician satisfaction of the IC workforce in particular [63]. Equally, financial considerations may bias institutional shareholder satisfaction away from investments in full-time programs, or physicians, with revenue streams that can be completely shut down by pandemic-related pressures. In the short term, they may prefer a more flexible model where temporary recruitment is made for a particular caseload during a COVID hiatus, rather than the exposure to longer term appointments who lose their luster if a COVID-only service returns. With interruptions to normal patterns of reward and recognition, climbing the traditional career ladder is extremely challenging at the present time.

The final workforce aspect applies to physician training. The pandemic severely disrupted training [64] and recruitment in 2020. The extent to which individuals will be adversely affected by these circumstances is beyond the scope of this article, but it would appear to be an important concept to try and envisage ways that would limit the impact of future waves of COVID-19 (or any future pandemic) on our trainees in particular. In a procedural specialty like interventional cardiology, this means that we have to fight to reset institutional priorities away from the obvious demands of the ICU, and cancer pathways in order to preserve operations in the CCL, and restore cardiovascular outcomes that at least resemble the pre-pandemic era.

## Conclusion

COVID-19 is now endemic. Infection spreads exponentially, and vaccination efforts are in their early stages. A high case fatality rate is expected for the typical cardiovascular patient demographic. During lulls in COVID infection, we must restructure our services and training, to sustain patient treatment while minimizing the burden on the hospital estate. We do this in order to make cardiovascular services in general, and CCL activity in particular, less vulnerable to waves of COVID transmission. The ICU model of care appears to be the safest for staff, but this comes at the greatest cost to the institution as beds are reduced, PPE is costlier, and staffing ratios are higher. In national healthcare systems that typically operate from single hospitals and run close to capacity, we may need to enter a phase where extended routine working hours into evening and weekends is needed to compensate for the reduced number of patients that can safely move though the hospital during regular working hours. At risk patients, told to isolate early in the pandemic, will be discerning customers of healthcare in the future. They will expect meticulous standards within hospitals and may be inclined to use digital health to seek out alternative providers from the comfort of their own living room. If the hospital cannot be maintained COVID-free in entirety, it should be at least COVID-free in the parts that need to remain open to treat COVID-vulnerable patients with otherwise treatable conditions. Many positive factors that contribute to physician well-being are compromised by the pandemic, but adversity does not come without opportunity. The opportunity of the present moment is to reconsider every aspect of cardiovascular medicine and accelerate transitions that may otherwise have taken a decade.
